# Band alignment and enhanced breakdown field of simultaneously oxidized and nitrided Zr film on Si

**DOI:** 10.1186/1556-276X-6-489

**Published:** 2011-08-10

**Authors:** Yew Hoong Wong, Kuan Yew Cheong

**Affiliations:** 1 Energy Efficient and Sustainable Semiconductor Research Group, School of Materials and Mineral Resources Engineering, Universiti Sains Malaysia, Engineering Campus, 14300 Nibong Tebal, Seberang Perai Selatan, Penang, Malaysia

**Keywords:** oxidation, sputtered-Zr, nitrous oxide, band alignment, electrical breakdown field

## Abstract

The band alignment of ZrO_2_/interfacial layer/Si structure fabricated by simultaneous oxidation and nitridation of sputtered Zr on Si in N_2_O at 700°C for different durations has been established by using X-ray photoelectron spectroscopy. Valence band offset of ZrO_2_/Si was found to be 4.75 eV, while the highest corresponding conduction offset of ZrO_2_/interfacial layer was found to be 3.40 eV; owing to the combination of relatively larger bandgaps, it enhanced electrical breakdown field to 13.6 MV/cm at 10^-6 ^A/cm^2^.

## Background

**Figure 1:**
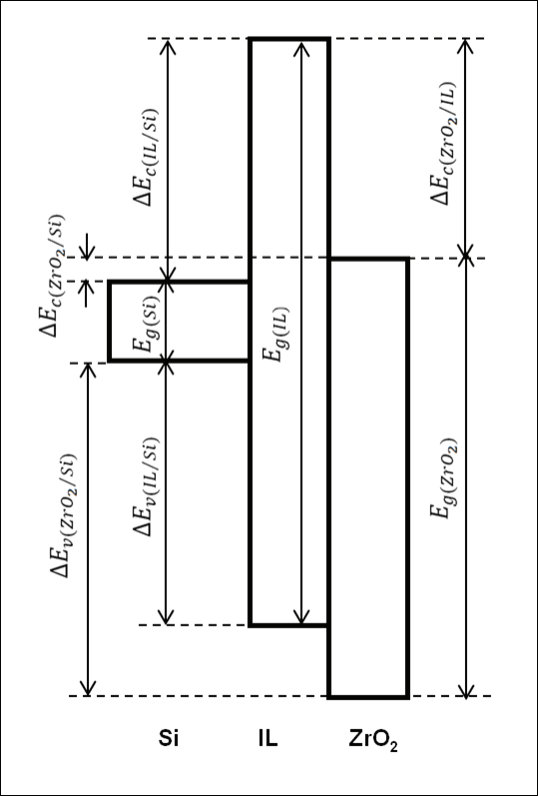
**Band alignment of ZrO_2_/IL/Si system**. *E*_g(ZrO2) _= bandgap of ZrO_2_, *E*_g(IL) _= bandgap of IL, *E*_g(Si) _= bandgap of Si, Δ*E*_v(ZrO2/Si) _= valence band offsets of ZrO_2 _to Si, Δ*E*_v(IL/Si) _= valence band offsets of IL to Si, Δ*E*_c(ZrO2/Si) _= conduction band offset of ZrO_2 _to Si, Δ*E*_c(IL/Si) _= conduction band offset of IL to Si, Δ*E*_c(ZrO2/IL) _= conduction band offset of ZrO_2 _to IL.

Application of high dielectric constant (*κ*) materials as future gate dielectrics on Si-based metal oxide semiconductor (MOS) devices has driven a tremendous research to realize an ultra-large-scale integrated circuitry with high performance and low power consumption [1-3]. Of various investigated high *κ *materials, ZrO_2 _is being considered as a potential gate dielectric for the near future generation technology nodes. It has been reported that excellent electrical properties of MOS capacitors that incorporated ZrO_2 _thin film as gate dielectric [4,5]. Putkonen *et al*. [5] and Niinisto *et al*. [4] have obtained the breakdown fields of ZrO_2 _at 6.0 and 9.5 MV/cm, respectively, at leakage current density of 10^-2 ^A/cm^2^. In order to attain excellent electrical properties of a device, interface properties of dielectric/Si play an indispensable role [6,7]. The leakage characteristic and electrical breakdown field of gate dielectric are basically dependent on the bandgap of the dielectric and on the band alignment with Si [8,9]. Hence, to use ZrO_2 _as gate dielectric in MOS capacitors, it should have sufficiently high band offsets with Si (> 1.00 eV) for both holes (valence band offset) and electrons (conduction band offset), so that an ultralow leakage current can be acquired [2,3]. Therefore, it is crucial to quantify these energy band offsets. Additionally, it is necessary to consider an interfacial layer (IL) that is inevitably formed in between ZrO_2 _and Si in the evaluation of band alignment. Works along this direction were reported by a number of researchers (Table 1). It is summarized that band alignment of the ZrO_2_/IL/Si system can be categorized into two types, depending on the oxide deposition techniques rather than the types (n or p) of semiconductor: type (i), alignment of ZrO_2 _bandgap in between the IL bandgap [10-13] and type (ii), alignment of ZrO_2 _conduction band outside the IL bandgap [14]. In this work, using simultaneous oxidation and nitridation of sputtered Zr on n-type Si in N_2_O, alignment of ZrO_2 _*valence band *outside the IL bandgap has been revealed [type (iii) in Table 1] (Figure 1). Owing to this type of alignment, dielectric electric breakdown field at low leakage current density has been enhanced.

**Table 1:** Comparison of the obtained values of *E*_g(ZrO2)_, *E*_g(IL)_, Δ*E*_v_, and Δ*E*_c_

Type	Deposition method	*E*_g(ZrO2)_	*E*_g(IL)_	Δ*E*_v_	Δ*E*_c_	Reference
(i)	Evaporation	5.50	8.60	1.00	1.90	[10]
	Sputtering	5.40	7.60	1.00	1.20	[11]
	Atomic layer chemical vapor deposition	5.80	7.60	1.15	1.05	[12]
	Electron beam deposition of Zr + oxidation in O_2_	5.80	9.00	1.80	1.40	[13]
(ii)	PLD	5.70	4.70	3.30 to 3.50	1.50	[14]
(iii)	Sputtering of Zr + oxidation and nitridation in N_2_O	6.20 to 6.50	8.20 to 8.80	4.75	3.40	This work

Type (i) defines alignment of ZrO_2 _bandgap in between the IL bandgap and type (ii) defines alignment of ZrO_2 _conduction band outside the IL bandgap. Type (iii) defines ZrO_2 _valence band outside the IL bandgap which is obtained from this work.

## Results and discussion

**Figure 2:**
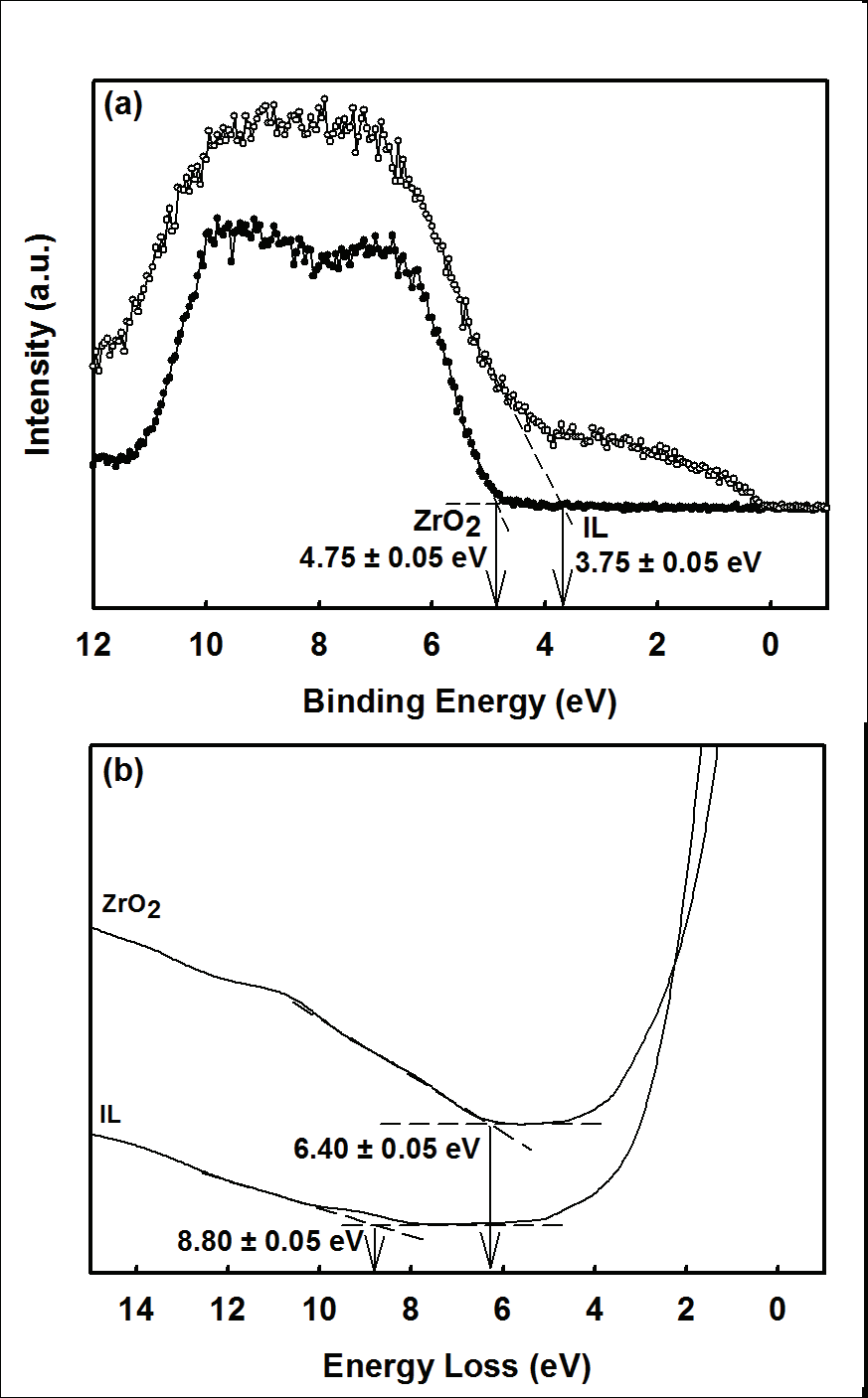
**XPS valence band spectra of ZrO_2 _and IL for all investigated samples**. (a) XPS valence band spectra of ZrO_2 _and IL for all investigated samples. (b) XPS O 1*s *plasmon loss spectra of ZrO_2 _and IL for 15-min sample.

**Figure 3:**
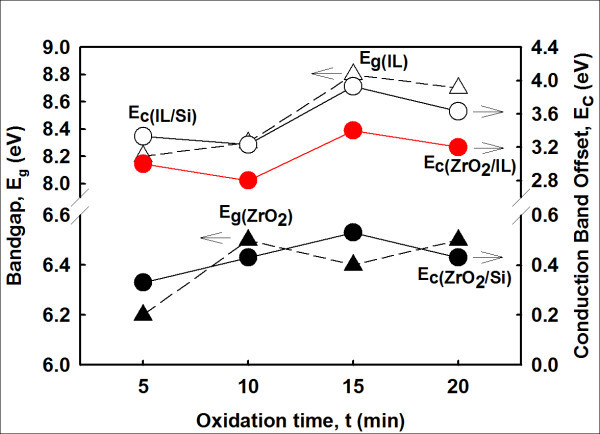
***E*_g _values of ZrO_2 _and IL extracted from their respective O 1*s *plasmon loss spectra**. The calculated values of *E*_g(ZrO2)_, *E*_g(IL)_, Δ*E*_c(ZrO2/Si)_, Δ*E*_c(IL/Si)_, and Δ*E*_c(ZrO2/IL) _in the band alignment of ZrO_2_/IL/Si system.

Figure 2a shows typical X-ray photoelectron spectroscopy (XPS) valence band spectra of ZrO_2 _and IL for all investigated samples. The valence band edges (*E*_v_) of ZrO_2 _and IL were estimated by an intercept of linear extrapolation of a maximum negative slope near the edge to the minimum horizontal baseline [10]. As a result, valence band offsets (Δ*E*_v_) of ZrO_2 _and IL with respect to Si substrate were 4.75 ± 0.05 eV and 3.75 ± 0.05 eV, respectively, for all investigated samples. To determine conduction band offset (Δ*E*_c_) of ZrO_2_/IL/Si system, the bandgaps (*E*_g_) of ZrO_2 _and IL were first deduced from O 1*s *plasmon loss spectra [15,16] of ZrO_2 _and IL, respectively. Figure 2b representatively demonstrates the XPS O 1*s *plasmon loss spectra of ZrO_2 _and IL for a 15-min sample. Whilst for other samples, the *E*_g _values of ZrO_2 _and IL extracted from their respective O 1*s *plasmon loss spectra are shown in Figure 3. As explained earlier, values of *E*_g _were also approximated by an intercept of linear extrapolation. The extracted *E*_g _values of ZrO_2 _and IL were 6.20 to 6.50 eV and 8.20 to 8.80 eV, respectively, with tolerance of 0.05 eV, dependent on the oxidation time (Figure 3). Ultimately, conduction band offset of ZrO_2 _to IL, Δ*E*_c(ZrO2/IL) _for the ZrO_2_/IL/Si system can be eventually derived [17]:(1)

where, *E*_g(ZrO2) _and *E*_g(IL) _are the bandgaps of ZrO_2 _and IL, respectively. Δ*E*_v(ZrO2/Si) _and Δ*E*_v(IL/Si) _are the valence band offsets of ZrO_2 _and IL, respectively, with respect to Si substrate. The calculated values of *E*_g(ZrO2)_, *E*_g(IL)_, Δ*E*_c(ZrO2/Si)_, Δ*E*_c(IL/Si)_, and Δ*E*_c(ZrO2/IL) _are presented in Figure 3. The highest value of Δ*E*_c(ZrO2/IL)_, i.e., 3.40 eV, was attained by sample oxidized/nitrided for 15 min (Figure 3) when compared to other samples. A schematic of the band alignment of the ZrO_2_/IL/Si system is illustrated in Figure 1. The *E*_g _value of Si substrate is obtained from literature [3,18]. It is found that values of *E*_g(ZrO2)_, *E*_g(IL)_, Δ*E*_c_, and Δ*E*_v _obtained in this study are higher than the values reported in literatures (Table 1).

**Figure 4:**
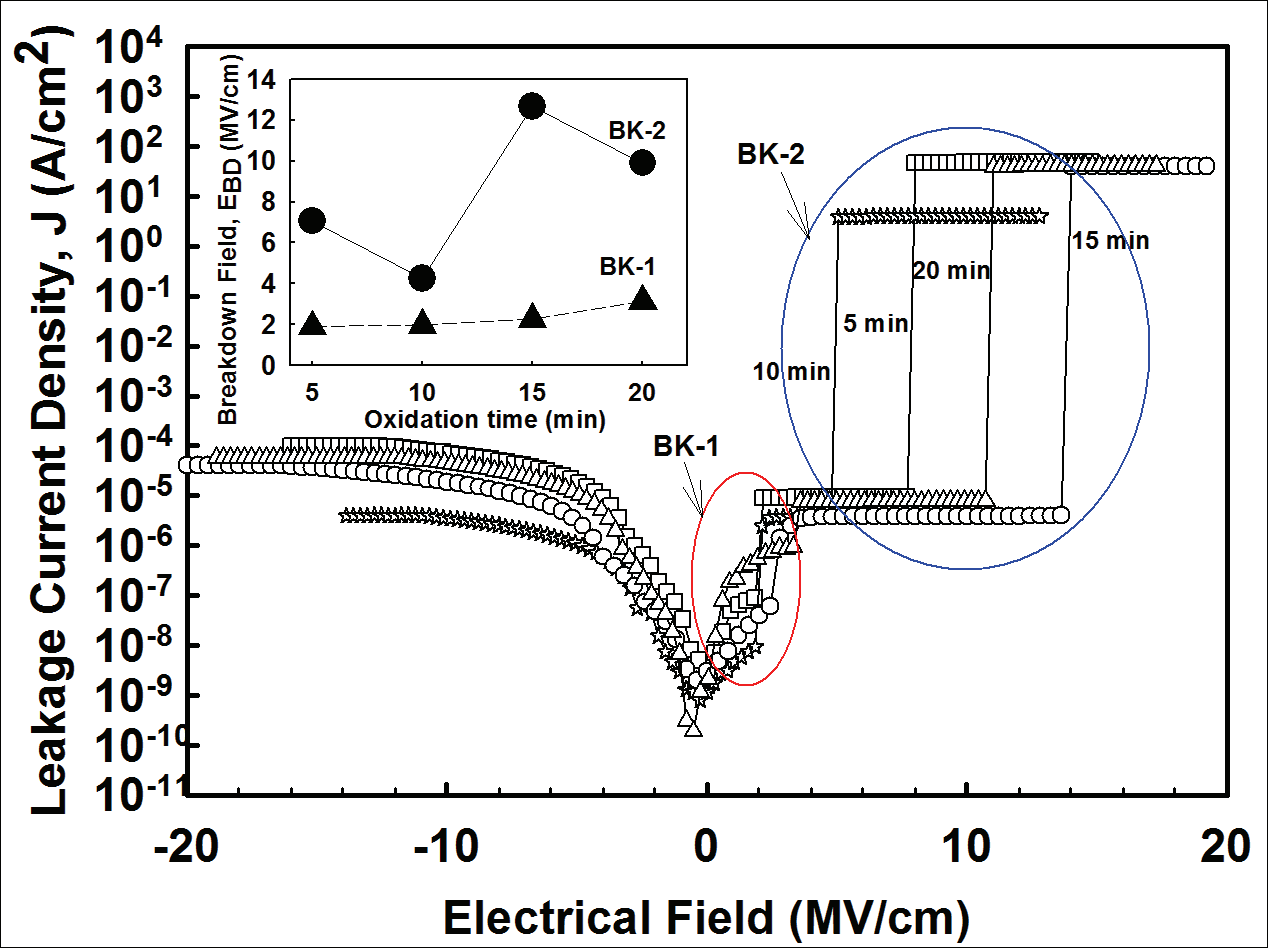
***J-E *characteristics of the investigated Al/ZrO_2_/IL/Si MOS capacitors**. Inset shows the average breakdown fields (BK-1 and BK-2) for the investigated samples.

Figure 4 shows typical leakage current density electric field (*J-E*) characteristics of the investigated samples. The *J-E *plot was transformed from current-voltage (*I-V*) measurement. The *E *value was estimated by first determining the flatband voltage (*V*_FB_) shift from the applied gate voltage (*V*_g_) and then dividing the total thicknesses of ZrO_2 _and IL (*t*_ox_) measured by energy filtered transmission electron microscopy (EFTEM) (images are not shown here). The acquired *J *value in this study is ~10^-8^A/cm^2 ^at *E *= 2.0 MV/cm, which is lower than the other studies [4,5,14].

A two-step oxide breakdown (BK-1 and BK-2) is being recorded in the *J-E *plot for all investigated samples (inset of Figure 4). The existence of interfacial and ZrO_2 _layers in the sample is the main cause of this two-step breakdown [19]. The breakdowns can be explained as follows. One of the layers may experience an electrical breakdown at a lower field, which is labeled as BK-1. Subsequently, another layer would block the carriers. Due to the increment of the electric field, the concentration of the carrier increases until the layer is electrically broken down at a higher electric field at BK-2. The instantaneous increment of leakage current density at BK-1 is relatively small when compared with others, and it is defined as soft breakdown. The magnitude of BK-1 increases gradually as the oxidation time is increased (inset of Figure 4). In contrast, the instantaneous increment of current density at BK-2 is large, and this is considered as hard breakdown. The highest dielectric breakdown field, which is referred to as hard breakdown, is attained by sample oxidized/nitride for 15 min (13.6 MV/cm at 10^-6 ^A/cm^2^). The lowest one is recorded by sample oxidized/nitride for 10 min (4.8 MV/cm at 10^-6 ^A/cm^2^). In comparison, dielectric breakdown field recorded in this work is higher than the previous reported works [4,5,14].

## Conclusions

In summary, the band alignment of ZrO_2_/IL/Si structure produced by simultaneous oxidation and nitridation of sputtered Zr thin film on Si in N_2_O has been established. Via this method, higher Δ*E*_c _and Δ*E*_v _values have been attained. Hence, a higher electrical breakdown field at low leakage current density has been achieved.

## Methods

The n-type Si(100) substrate with a resistivity of 1 to 10 Ω cm was used in this study. After undergoing a standard wafers cleaning process, a 5-nm thick Zr film was sputtered on the cleaned Si substrates by an RF sputtering system. Following that, samples were loaded into a horizontal tube furnace and were heated up from room temperature to 700°C in an Ar flow ambient, and the heating rate was fixed at 10°C/min. Once the set temperature was achieved, N_2_O gas was introduced with a flow rate of 150 mL/min for a set of durations (5, 10, 15, and 20 min). After the furnace was cooled down to room temperature in an Ar ambient, the samples were withdrawn from the furnace. To experimentally determine band alignment of the dielectric/semiconductor structure, XPS measurements were conducted using Kratos Axis Ultra DLD (Kratos Analytical, Chestnut Ridge, NY, USA). with a monochromatic Al-*K_α _*X-ray source (hν = 1,486.69 eV) performed at the Research Center for Surface and Materials Science, The Auckland University, New Zealand. The spectra of survey or wide scan (binding energy of -5 to 25 eV) were collected at a take off angle of 0° with respect to surface normal, with low pass energy of 20 eV and small step size of 0.1 eV. Due to the onset of single particle excitation and band-to-band transition, the energy loss spectrum of O 1*s *photoelectron provides further insight on the bandgaps of ZrO_2 _and IL [20]. Subsequently, a detail scan of O 1*s *was carried out using the same pass energy and step size of 1.0 eV. Ar ion gun (5 keV) was employed to etch the sample in order to perform chemical depth profiling (results are not shown here), in order for the boundary of ZrO_2 _and IL to be identified. A Shirley background function, which is proportional to the integrated photoelectron peak area, was subtracted from all of the XPS spectra to correct for the inelastic photoelectron scattering effect [21]. Band alignment extraction was based on Kraut method [15,16]. As to characterize the leakage characteristic and electrical breakdown field of the film, MOS capacitor test structure was formed by thermally evaporated a 100-nm thick aluminum (Al) film, acting as a gate electrode, on top of the films. The area of a capacitor was photolithographically defined at 9 × 10^-4 ^cm^2^. In order to obtain an Ohmic back contact, a 100-nm thick Al film was thermally evaporated on the backside of the Si substrate after removal of native oxide. *I-V *measurements were performed by a computer-controlled Agilent HP4155-6C semiconductor parameter analyzer (Agilent Technologies, Santa Clara, CA, USA).

## Competing interests

The authors declare that they have no competing interests.

## Authors' contributions

YHW has been involved in the experimental design, data acquisition, data interpretation and analysis, and drafting and revision of the manuscript. KYC has been involved in revising the manuscript critically for important intellectual content and has given final approval to the version to be submitted for publication.

## References

[B1] Robertson J High dielectric constant oxides Eur Phys J Appl Phys 2004 28 265 291 10.1051/epjap:2004206

[B2] Wilk GD Wallace RM Anthony JM High-k gate dielectrics: current status and materials properties considerations J Appl Phys 2001 89 5243 5275 10.1063/1.1361065

[B3] Wong YH Cheong KY ZrO_2 _thin films on Si substrate J Mater Sci: Mater Electron 2010 21 980 993 10.1007/s10854-010-0144-5

[B4] Niinisto J Putkonen M Niinisto L Kukli K Ritala M Leskela M Structural and dielectric properties of thin ZrO_2 _films on silicon grown by atomic layer deposition from cyclopentadienyl precursor J Appl Phys 2004 95 84 91 10.1063/1.1630696

[B5] Putkonen M Niinistö J Kukli K Sajavaara T Karppinen M Yamauchi H Niinistö L ZrO_2 _thin films grown on silicon substrates by atomic layer deposition with Cp_2_Zr(CH_3_)_2 _and water as precursors Chem Vap Dep 2003 9 207 212 10.1002/cvde.200306254

[B6] Aygun G Yildiz I Interfacial and structural properties of sputtered HfO_2 _layers J Appl Phys 2009 106 014312 014317 10.1063/1.3153953

[B7] Weinreich W Ignatova VA Wilde L Teichert S Lemberger M Bauer AJ Reiche R Erben E Heitmann J Oberbeck L Schroder U Influence of N_2 _and NH_3 _annealing on the nitrogen incorporation and k-value of thin ZrO_2 _layers J Appl Phys 2009 106 034107 034107 10.1063/1.3187829

[B8] Chen Q Feng YP Chai JW Zhang Z Pan JS Wang SJ Band alignment and thermal stability of HfO_2 _gate dielectric on SiC Appl Phys Lett 2008 93 052104 052103 10.1063/1.2969061

[B9] Yim CJ Ko DH Jang MH Chung KB Cho MH Jeon HT Change in band alignment of HfO_2 _films with annealing treatments Appl Phys Lett 2008 92 012922 012923 10.1063/1.2826270

[B10] Miyazaki S Characterization of high-k gate dielectric/silicon interfaces Appl Surf Sci 2002 190 66 74 10.1016/S0169-4332(01)00841-8

[B11] Houssa M Tuominen M Naili M Afanas'ev V Stesmans A Haukka S Heyns MM Trap-assisted tunneling in high permittivity gate dielectric stacks J Appl Phys 2000 87 8615 8620 10.1063/1.373587

[B12] Afanas'ev VV Houssa M Stesmans A Heyns MM Band alignments in metal-oxide-silicon structures with atomic-layer deposited Al_2_O_3 _and ZrO_2_ J Appl Phys 2002 91 3079 3084 10.1063/1.1436299

[B13] Fulton CC Cook JTE Lucovsky G Nemanich RJ Interface instabilities and electronic properties of ZrO_2 _on silicon (100) J Appl Phys 2004 96 2665 2673 10.1063/1.1776313

[B14] Yamaguchi T Satake H Fukushima N Band diagram and carrier conduction mechanisms in ZrO_2 _MIS structures IEEE Trans on Electron Devices 2004 51 774 779 10.1109/TED.2004.826973

[B15] Kraut EA Grant RW Waldrop JR Kowalczyk SP Precise determination of the valence band edge in x-ray photoemission spectra: application to measurement of semiconductor interface potentials Phys Rev Lett 1980 44 1620 10.1103/PhysRevLett.44.1620

[B16] Kraut EA Grant RW Waldrop JR Kowalczyk SP Semiconductor core-level to valence band maximum binding-energy differences: precise determination by x-ray photoelectron spectroscopy Phys Rev B 1983 28 1965 10.1103/PhysRevB.28.1965

[B17] Zhu LQ Zhang LD Li GH He G Liu M Fang Q Thermal stability and energy-band alignment of nitrogen-incorporated ZrO_2 _films on Si(100) Appl Phys Lett 2006 88 232901 232903 10.1063/1.2209882

[B18] Muller RS Kamins TI Device Electronics for Integrated Circuits 1986 2 54

[B19] Xiaolong Y Qianghua X Tao M Electrical breakdown in a two-layer dielectric in the MOS structure Mat Res Soc Symp Proc 2004 811 D2.8.1

[B20] Miyazaki S Nishimura H Fukuda M Ley L Ristein J Structure and electronic states of ultrathin SiO_2 _thermally grown on Si(100) and Si(111) surfaces Appl Surf Sci 1997 113-114 585 589

[B21] Shirley DA High-resolution x-ray photoemission spectrum of the valence bands of gold Phys Rev B 1972 5 4709 10.1103/PhysRevB.5.4709

